# A Retrospective Cohort Study of Hospitalised Patients With First‐Episode Substance‐Induced Psychosis and First‐Episode Psychosis: Comparing Long‐Acting Injectable Antipsychotic Use and Post‐Discharge Outcomes

**DOI:** 10.1111/eip.70250

**Published:** 2026-09-21

**Authors:** John D. Eun, Matt Iles‐Shih, Suelynn Ren, Kevin A. Hallgren, Adam Kuczynski

**Affiliations:** ^1^ Department of Psychiatry and Behavioral Sciences University of Washington School of Medicine Seattle Washington USA

**Keywords:** antipsychotics, first‐episode psychosis, hospitalisation, long‐acting injectable, substance use, substance‐induced psychoses

## Abstract

**Introduction:**

Clinicians may not recommend long‐acting injectable antipsychotics (LAI‐APs) for patients diagnosed with first‐episode substance‐induced psychosis (FESIP), despite the risk of transition from FESIP to schizophrenia and the effectiveness of LAI‐AP in early psychosis. We compared LAI‐AP use and post‐discharge outcomes between patients with first‐episode psychosis (FEP) and those with FESIP during their index psychiatric hospitalisation.

**Methods:**

We conducted a retrospective cohort study using electronic health record data from patients aged 18–39 who were discharged from inpatient psychiatry units at a public academic hospital between 2021 and 2024. Outcomes included administration of any LAI‐AP during the index hospitalisation, documented recommendation of any LAI‐AP, psychiatric rehospitalisation and emergency department (ED) encounters after discharge.

**Results:**

The cohort included 90 patients with FEP and 84 patients with FESIP. Only one (1.2%) patient with FESIP received an LAI‐AP compared with 10 (11.1%) with FEP (OR = 0.10, 95% CI = 0.002–0.71); LAI‐AP was recommended to one (1.2%) patient with FESIP compared with 30 (33.3%) with FEP (OR = 0.02, 95% CI = 0.0006–0.16). Rehospitalisation occurred in 41 (45.6%) patients with FEP and 38 (45.2%) with FESIP; post‐discharge ED encounters occurred in 50 (55.6%) and 52 (61.9%) patients, respectively. Time to first rehospitalisation and ED encounter after discharge did not differ between the groups.

**Conclusions:**

Despite similar post‐discharge outcomes between patients with FEP and FESIP, administration or recommendation of an LAI‐AP was rarer among those with FESIP. Our findings highlight uncertainty in treatment decision‐making and support further research to guide early intervention strategies in patients with substance‐induced psychosis.

## 1 Introduction

Individuals who are hospitalised for their first psychotic episode in the context of substance use present a diagnostic challenge. Substance use is prevalent among individuals who are diagnosed with a primary first‐episode psychosis (FEP) that may be due to chronic schizophrenia spectrum disorders (SSDs) such as schizophrenia (Addington and Addington 2007; Brunette et al. 2018). On the other hand, because substance use can ‘induce’ episodes of psychosis that last beyond the initial period of intoxication, clinicians may diagnose individuals presenting with their first psychotic episode as having substance‐induced psychosis (SIP). Clinicians differentiate first‐episode SIP (FESIP) from FEP by considering various demographic, familial, and clinical characteristics, including the temporal correlation with substance use, age of onset, family history of psychosis and the presence of prodromal or negative symptoms (Caton et al. 2005). Yet, a substantial proportion of those with FESIP may transition to chronic SSDs. A meta‐analysis found that the pooled rate of transition was 25% for SIP compared with 36% for brief, atypical and unspecified psychoses (Murrie et al. 2020).

For both FEP and FESIP, antipsychotic medications are effective in reducing symptoms and preventing relapse (Farnia et al. 2014; Chuenchom et al. 2024; Mustonen et al. 2025). But approximately one third of individuals with their first episode of schizophrenia stopped taking their antipsychotic medication within 30 days of discharge (Tiihonen et al. 2018), and substance use further interferes with medication adherence (Schoeler et al. 2017). Long‐acting injectable antipsychotics (LAI‐APs) have been associated with improved adherence and a lower risk of rehospitalisation compared with oral antipsychotics (oral‐APs) among those with FEP (with or without co‐morbid substance use disorder) or early‐phase schizophrenia (Abdel‐Baki et al. 2020; Kane et al. 2020; Denissoff et al. 2024). LAI‐AP use also appears to be associated with the lowest risk of relapse for individuals with cannabis‐induced psychosis (Mustonen et al. 2025).

However, patients diagnosed with FESIP may receive suboptimal treatment planning in the inpatient psychiatry setting (Rognli, Heiberg, et al. 2023). One retrospective study of individuals hospitalised on inpatient psychiatry units found a disproportionately low rate of referral to early intervention programmes for those diagnosed with FESIP (4.3%) versus FEP with concurrent substance use (53.8%), even though a third of all patients with FESIP transitioned to chronic SSDs compared with half of those with FEP (Inchausti et al. 2023). Similarly, clinicians may be less likely to recommend an LAI‐AP to those with FESIP because they perceive the risk of rehospitalisation to be low as long as patients abstain from substance use. As the risk of transition from FESIP to chronic SSDs may be clinically meaningful, those with FESIP might be at greater risk of negative post‐discharge outcomes if they are less likely to receive effective treatments. In this retrospective study of individuals who were hospitalised for their first psychotic episode, we evaluated whether the likelihood of receiving an LAI‐AP and negative post‐discharge outcomes differed between patients diagnosed with FEP and those with FESIP. We hypothesised that patients diagnosed with FESIP would be less likely to receive an LAI‐AP and would be at greater risk of post‐discharge rehospitalisation and ED encounter than those with FEP.

## 2 Methods

We preregistered our hypotheses, data analysis plan and inference criteria on the Open Science Framework (https://osf.io/ezrhm).

### 2.1 Study Sample

We analysed electronic health record (EHR) data of people who were discharged from three inpatient psychiatry units at Harborview Medical Center (HMC) in Seattle, WA, a public academic tertiary/quaternary care facility that serves as a Level I trauma centre for four states (Washington, Alaska, Idaho, Montana) in the United States. The University of Washington Institutional Review Board approved the study prior to accessing the data. We reviewed 3175 unique patients who were discharged between 1 January 2021 and 31 December 2024 for study eligibility. From the dataset, we identified a cohort of individuals who were diagnosed with their first known episode of psychosis during their index hospitalisation. Patients were included if they were aged 18–39 and excluded if their first psychotic episode was due to a primary mood disorder or personality disorder.

### 2.2 Data Collection

The primary reviewer (JDE) reviewed the EHR records to determine eligibility and collect data, including documentation from HMC encounters and from other hospitals when accessible through the EHR's health information exchange. In cases where the diagnosis was equivocal (e.g., first episode vs. recurrent episode, FEP vs. FESIP), a second reviewer (S.R.) independently reviewed the EHR to assign the diagnosis. If the two reviewers (J.D.E., S.R.) disagreed, a third reviewer (M.I.‐S.) served as the tiebreaker.

### 2.3 Variables

Our sample consisted of two groups: patients who were diagnosed with SIP for the first time (i.e., FESIP) and those who were diagnosed with FEP during the index hospitalisation. We defined ‘LAI‐AP use’ when patients received any long‐acting injectable antipsychotic during the index hospitalisation. We also assessed the treatment team's intent to administer an LAI‐AP by determining whether they documented recommending an LAI‐AP to the patient prior to discharge (‘LAI‐AP intent’). If the clinician documented consideration of an LAI‐AP but did not document a conversation about LAI‐AP with the patient during their hospitalisation, LAI‐AP intent was coded ‘no’. This excluded documentation that was contingency‐based, capturing cases in which clinicians discussed LAI‐APs as reasonable alternatives to oral‐APs during informed consent.

We used a hierarchical categorisation of substance use because cannabis and illicit stimulants (i.e., excluding prescribed stimulants) are most frequently associated with substance‐induced psychosis (Rognli, Taipale, et al. 2023). If a patient used both cannabis and illicit stimulants such as methamphetamine, we categorised their use as concurrent cannabis and stimulant use (+THC/+Stim). If a patient used cannabis but not illicit stimulants, or illicit stimulants but not cannabis, we categorised their use as any cannabis use (+THC/−Stim) or any stimulant use (−THC/+Stim), respectively, regardless of the use of other substances such as alcohol or hallucinogens.

We determined rehospitalisation and ED encounter by reviewing all UW Medicine and non‐UW Medicine hospital and outpatient encounters after the index hospitalisation at HMC. We collected information on the date of the first rehospitalisation or first ED encounter for psychotic relapse following discharge as well as the number of such events within 30 days of discharge.

### 2.4 Statistical Analysis

Group differences in sociodemographic and clinical characteristics were evaluated using *χ* ^2^ tests for categorical variables, or Fisher's exact test when expected cell counts were too small to meet *χ* ^2^ test assumptions. For continuous variables, we used *t*‐tests and calculated standardised mean differences (SMDs). We planned to use a modified Poisson regression model to test for differences in the probability of receiving an LAI‐AP between patients with FEP and FESIP, while adjusting for baseline characteristics, family history of psychosis, any cannabis use and the interaction between diagnosis and any cannabis use. Modified Poisson regression was used to compare the probability of 30‐day rehospitalisation between patients with FEP and those with FESIP. Cox proportional hazards models were used to estimate time to post‐discharge rehospitalisation and first ED encounter during the study follow‐up period (from 1 January 2021 to 30 June 2025). When Cox proportional hazards model assumptions were not met, Poisson regression was used as an alternative. Sensitivity analyses assessed the impact of adjusting for covariates with significant baseline differences, missing family history data and the inclusion of cases with psychosis onset within 3 years prior to the index hospitalisation. All analyses were conducted using R 4.4.2 software (R Core Team 2024).

## 3 Results

We identified 174 patients who were diagnosed with either FEP (*n* = 90) or FESIP (*n* = 84) during their index hospitalisation (Table 1). Compared with patients with FEP, those with FESIP were, on average, older (FEP 25.84 vs. FESIP 28.75 years, *p =* 0.002) and had a lower rate of involuntary hospitalisation (FEP 85.6% vs. FESIP 64.3%, *p =* 0.002). All but eight patients used cannabis or stimulants; Table 1 therefore does not present data on other substances. Any cannabis use was the most common (110/174, 63.2%), followed by any illicit stimulant use (47/174, 27.0%). We found differences in the rates of any stimulant use (FEP 5.6% vs. FESIP 21.4%, *p* = 0.004) and concurrent cannabis and stimulant use (FEP 2.2% vs. FESIP 26.2%, *p <* 0.001). Antipsychotic medications were prescribed on discharge more often for patients with FEP (*n* = 85, 94.4%) compared with patients with FESIP (*n* = 63, 75.0%; *p <* 0.001). Notably, family history of psychosis was not documented for 26.7% and 36.9% of patients with FEP and FESIP, respectively.

**TABLE 1:** Baseline characteristics of patients with first‐episode psychosis compared with those with first‐episode substance‐induced psychosis during their index hospitalisation.

Socio‐demographics	FEP (*n* = 90)	FESIP (*n* = 84)	*p*
Age, mean (SD)	25.84 (5.47)	28.75 (6.47)	0.002
Male gender, *n* (%)	60 (66.7)	64 (76.2)	0.22
Race			
Non‐Hispanic White, *n* (%)	28 (31.1)	36 (42.9)	0.067
Non‐Hispanic Black, *n* (%)	34 (37.8)	19 (22.6)	
Asian, *n* (%)	16 (17.8)	11 (13.1)	
Other, *n* (%)	12 (13.3)	18 (21.4)	
Housing instability, *n* (%)	19 (21.1)	18 (21.4)	1.00

Clinical measures	FEP (*n* = 90)	FESIP (*n* = 84)	*p*
Family psychiatric history, *n* (%)	41 (45.6)	35 (41.7)	0.11
Family history of psychosis,^a^ *n* (%)	19 (21.1)	12 (14.3)	0.26
Substance use, *n* (%)	57 (63.3)	84 (100)	< 0.001
Any cannabis, *n* (%)	46 (51.1)	40 (47.6)	0.76
Any stimulant, *n* (%)	5 (5.6)	18 (21.4)	0.004
Concurrent cannabis and stimulant, *n* (%)	2 (2.2)	22 (26.2)	< 0.001
Involuntary hospitalisation, *n* (%)	77 (85.6)	54 (64.3)	0.002
Behavioural health referral, *n* (%)	49 (54.4)	44 (52.3)	0.87
Antipsychotic on discharge, *n* (%)	85 (94.4)	83 (75.0)	< 0.001
Oral antipsychotic only, *n* (%)	75 (83.3)	62 (73.8)	0.18
LAI‐AP, *n* (%)	10 (11.1)	1 (1.2)	0.01^b^
LAI‐AP intent, *n* (%)	30 (33.3)	1 (1.2)	< 0.0001^b^

Clinical measures	FEP (*n* = 90)	FESIP (*n* = 84)	SMD
Length of hospitalisation, mean (SD)	19.47 (14.48)	9.29 (6.08)	−0.83^c^
Antipsychotic TDD, mean (SD)	13.40 (7.51)	11.67 (6.50)	−0.24^d^

Abbreviations: FEP, first‐episode psychosis; FESIP, first‐episode substance‐induced psychosis; LAI‐AP, long‐acting injectable antipsychotic; SD, standard deviation; SMD, standardised mean difference; TDD, total daily dose.

^a^ Family history of psychosis was missing for 26.7% and 36.9% of patients with FEP and FESIP, respectively.

^b^ Fisher's exact test was applied.

^c^ *p* < 0.001.

^d^ *p* = 0.13.

### 3.1 LAI‐AP Use and LAI‐AP Intent

During the index hospitalisation, 10 (11.1%) patients with FEP and one patient (1.2%) with FESIP received an LAI‐AP (Table 1). Because only one patient with FESIP received an LAI‐AP, we could not evaluate the association between diagnosis group (FESIP vs. FEP) and LAI‐AP use or LAI‐AP intent with our preregistered Poisson regression analysis that controlled for covariates. In an unadjusted comparison using Fisher's exact test, patients with FESIP had between 29% and 99.8% lower odds of receiving an LAI‐AP than those with FEP (OR = 0.10, 95% CI = 0.002–0.71) prior to discharge. Intent to administer LAI‐AP was documented for only one (1.2%) patient with FESIP compared with 30 (33.3%) patients with FEP. Fisher's exact test showed that patients with FESIP had between 84% and 99.94% lower odds of LAI‐AP intent than those with FEP (OR = 0.02, 95% CI = 0.0006–0.16).

#### 3.1.1 Rehospitalisation After Discharge

**FIGURE 1:**
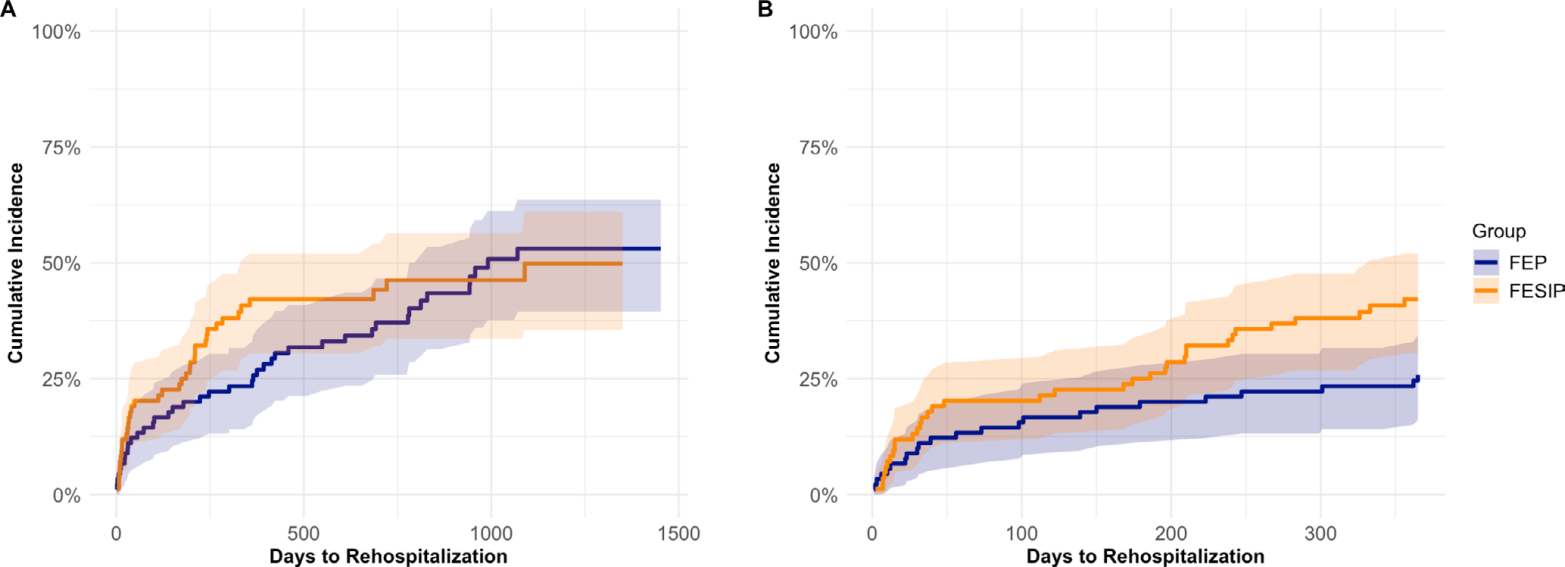
Cumulative incidence of rehospitalisation (A) during the follow‐up period (January 2021–June 2025) and (B) within 1‐year post‐discharge. Abbreviations: FEP, first‐episode psychosis; FESIP, first‐episode substance‐induced psychosis.

During follow‐up through 30 June 2025, rehospitalisation occurred in 41 (45.6%) patients with FEP and 38 (45.2%) patients with FESIP, with median time to first rehospitalisation (among those who were rehospitalised) of 301 days for FEP and 145 days for FESIP. Visual inspection of Kaplan–Meier plots indicated uncertainty as to whether the cumulative incidence of rehospitalisation was different between the groups over time (Figure 1A). We found no difference between patients with FEP and those with FESIP in time to rehospitalisation (HR = 1.07, 95% CI 0.66–1.73; Table 2) or risk of 30‐day rehospitalisation (RR = 1.70, 95% CI 0.61–4.73; Table S1). Because the former did not meet the proportional hazards assumptions, we conducted a non‐preregistered Poisson regression model and found no significant effect of diagnosis on the overall risk of rehospitalisation (RR = 1.21, 95% CI = 0.70–2.08; Table S2). As the Kaplan–Meier curve showed that the cumulative incidence of rehospitalisation for FESIP initially increased at a greater rate than that of FEP before stabilising (Figure 1A,B), we conducted post hoc analyses limiting the follow‐up period to 1 year with a Cox proportional hazards model, which met the proportional hazards assumptions. Compared with patients with FEP, the cumulative hazard of 1‐year rehospitalisation for those with FESIP was as low as 1% less likely and as high as 212% more likely (HR = 1.75, 95% CI = 0.99–3.12; Table 2).

**TABLE 2:** Hazard ratios from Cox proportional hazards models for time to rehospitalisation during follow‐up, time to rehospitalisation within 1‐year post‐discharge and time to ED encounter during follow‐up, according to diagnosis and other covariates.

	Rehospitalisation during follow‐up		Rehospitalisation within 1 year		ED encounter during follow‐up	
	HR	95% CI	HR	95% CI	HR	95% CI
FESIP	1.07	0.66–1.73	1.75	0.99–3.12	1.32	0.87–2.01
LAI‐AP use	0.67	0.21–2.18	0.62	0.15–2.62	1.08	0.43–2.70
Age	1.00	0.96–1.04	0.99	0.94–1.03	1.01	0.98–1.05
Male	1.05	0.63–1.74	1.18	0.63–2.20	1.20	0.76–1.88
Any cannabis use	1.30	0.78–2.17	0.98	0.53–1.79	1.02	0.66–1.59

Abbreviations: CI, confidence interval; ED, emergency department; FESIP, first‐episode substance‐induced psychosis; HR, hazard ratio; LAI‐AP, long‐acting injectable antipsychotic.

#### 3.1.2 ED Encounter After Discharge

**FIGURE 2:**
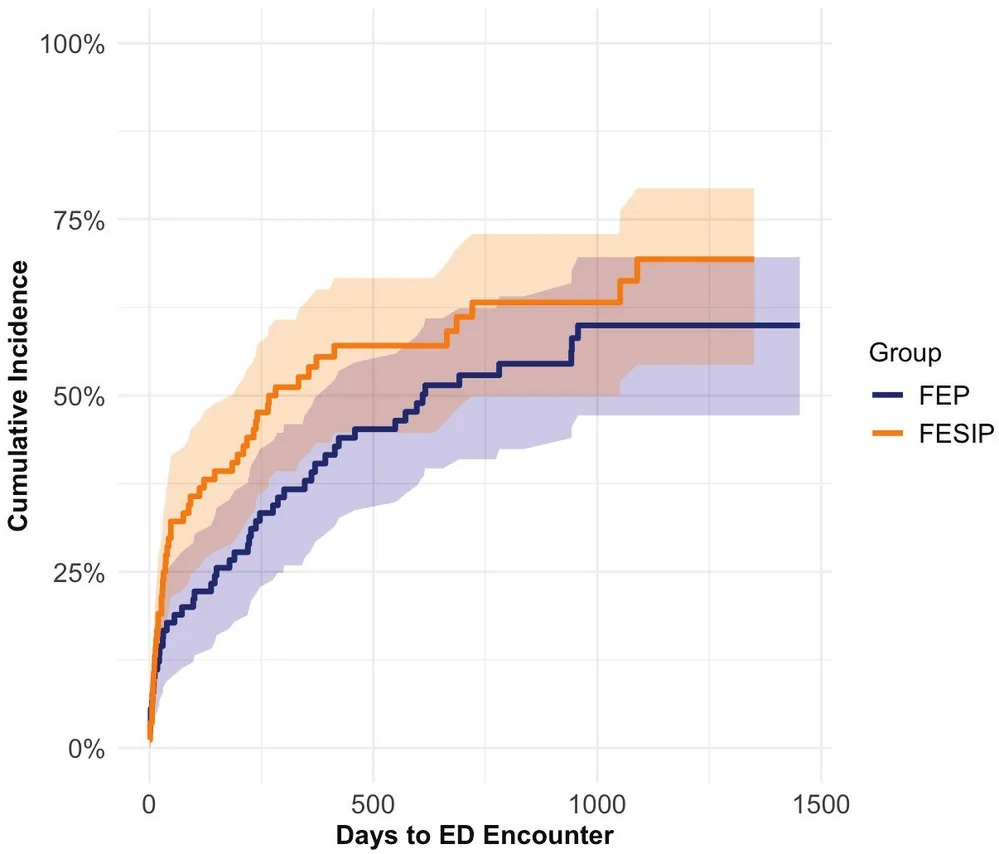
Cumulative incidence of ED encounter post‐discharge during the follow‐up period (January 2021–June 2025). Abbreviations: FEP, first‐episode psychosis; FESIP, first‐episode substance‐induced psychosis.

Post‐discharge ED encounters occurred in 50 (55.6%) patients with FEP and 52 (61.9%) patients with FESIP, with median time to first encounter of 205 and 48 days, respectively. Kaplan–Meier plots showed convergence of cumulative incidence over time with overlapping confidence intervals throughout the follow‐up period (Figure 2). We found no difference between patients with FEP and those with FESIP in time to first ED encounter (HR = 1.32, 95% CI = 0.87–2.01; Table 2). There was no difference between patients with FEP and those with FESIP in the risk of 30‐day ED encounter (RR = 1.78, 95% CI = 0.81–3.95; Table S2).

### 3.2 Sensitivity Analyses

Sensitivity analyses adjusting for race/ethnicity and involuntary hospitalisation status did not meaningfully change post‐discharge outcome estimates (Table S3A,B). When we expanded the sample to include patients who had experienced psychosis within 3 years prior to their index hospitalisation (FEP3Y, *n* = 161; FESIP3Y, *n* = 143), LAI‐AP use during the index hospitalisation, time to rehospitalisation, or risk of 30‐day rehospitalisation did not differ between FESIP3Y and FEP3Y (Table S4A–C), but patients with FESIP3Y were more likely to have an ED encounter at any given time than those with FEP3Y (HR = 1.53, 95% CI = 1.14–2.07).

## 4 Discussion

This retrospective cohort study compared long‐acting injectable antipsychotic (LAI‐AP) use and post‐discharge outcomes between patients diagnosed with first‐episode psychosis (FEP) and those with first‐episode substance‐induced psychosis (FESIP) during their index psychiatric hospitalisation. In unadjusted analyses, patients with FESIP had markedly lower probabilities of receiving an LAI‐AP (‘LAI‐AP use’) and having a documented recommendation of an LAI‐AP (‘LAI‐AP intent’) than those with FEP, although most patients with FEP and FESIP were discharged with oral antipsychotics. However, the preregistered analyses adjusting for potential confounders were not feasible because only a single patient with FESIP received or was recommended an LAI‐AP.

Several factors may contribute to the relatively low probabilities of both LAI‐AP use and LAI‐AP intent among patients with FESIP in our sample. Clinicians may conceptualise FESIP as a transient condition that remits with abstinence (even without long‐term medications) or determine that the risk–benefit analysis for improving medication adherence with an LAI‐AP is less favourable in FESIP than in FEP. Although the shorter length of hospitalisation among patients with FESIP could reflect more rapid symptom resolution with abstinence, it may also indicate that clinicians are less willing to extend a patient's hospitalisation to establish outpatient support for maintenance LAI‐AP. These interpretations align with a previous qualitative study showing that clinicians often consider LAI‐APs as a late‐stage intervention for individuals with recurrent relapses due to nonadherence (Iyer et al. 2013).

This work also highlights methodological challenges in using retrospective EHR data to evaluate clinical decision‐making related to LAI‐APs in the inpatient context. Our ability to measure clinicians' intentions to administer LAI‐APs depended on consistent documentation of recommending an LAI‐AP, but documentation practices may have varied without specific policies that could increase consistency (e.g., establishing LAI‐AP administration as a quality metric). Actual administration of LAI‐AP was objectively measured but influenced by several factors. In addition to patient‐level acceptability—frequently characterised by dislike of injections—clinicians may vary in how they introduce an LAI‐AP and how they respond to a patient's hesitancy (Potkin et al. 2013). Structural barriers such as health insurance constraints might have also influenced treatment decisions.

Moreover, we found no difference in the likelihood of rehospitalisation or ED encounters for those with FEP versus FESIP. This finding is consistent with prior evidence suggesting that some patients with FESIP may experience a recurrent or persistent course (Murrie et al. 2020; Rognli, Heiberg, et al. 2023). When we expanded the sample to include those with psychosis onset within 3 years prior to the index hospitalisation, patients with FESIP within 3 years were more likely to have an ED encounter at any given time than those with FEP within 3 years. This elevated risk may reflect acute presentations related to stimulant intoxication or opioid overdose, given the higher prevalence of stimulant and non‐prescription opioid use in this group.

These findings raise the question of whether evidence‐based guidelines for FEP should also inform the management of FESIP. Although LAI‐AP use was not associated with post‐discharge outcomes in this study, the small number of LAI‐AP users limits the study's ability to detect associations between LAI‐AP use and post‐discharge outcomes and should be interpreted alongside existing evidence on LAI‐AP for early psychosis. The latest international guideline for treatment of schizophrenia recommends that clinicians consider an LAI‐AP as one of the treatment options for FEP, given its benefits for improving adherence, reducing risk of relapse and lowering overall mortality (McCutcheon et al. 2025). The guideline advises caution when substance use is present, possibly because evidence on LAI‐AP for FESIP is sparse and largely derived from observational studies in similar populations. Such studies have shown that LAI‐APs are associated with a lower risk of psychotic relapse when compared with oral antipsychotics for those with either FEP with co‐occurring substance use disorder or cannabis‐induced psychosis (Abdel‐Baki et al. 2020; Denissoff et al. 2024; Mustonen et al. 2025). The present study contributes to the growing literature that underscores the need for interventional research to determine whether LAI‐AP could mitigate both the short‐term risk of rehospitalisation and the long‐term risk of transition to chronic forms of psychosis for patients with FESIP.

This study has several limitations. While the hospital from which we sampled serves as both the safety‐net hospital for an urban population and Level I trauma centre for the region, it is also an academic facility where psychiatric pharmacists may influence treatment decisions as members of patients' treatment teams. Consequently, our single‐site setting limits generalisability, especially for populations that face fewer structural barriers to accessing LAI‐APs or outpatient resources than our study population and for rural, community hospitals. Our process for equivocal diagnoses may limit external validity, as such cases might remain diagnostically ambiguous in clinical practice. Although these cases were uncommon in our sample, future studies should consider that such cases may represent a clinically meaningful subgroup in the broader population. We were limited to rehospitalisation and ED encounter data for health systems that share data through a health information exchange, likely leading to some underestimation of these outcomes. Even though sensitivity analyses suggested minimal impact, missing data on family history of psychosis may have also introduced bias. Some preregistered analyses did not meet modelling assumptions, and the post hoc nature of non‐preregistered analyses warrants cautious interpretation.

## 5 Conclusion

Patients with FESIP were significantly less likely to receive or be recommended an LAI‐AP during their psychiatric hospitalisation than those with FEP in unadjusted analyses. Because only a small proportion of patients with FEP or FESIP received an LAI‐AP, estimates of the association between LAI‐AP use and post‐discharge outcomes were imprecise. Nonetheless, the two groups had similar cumulative incidences of rehospitalisation and ED encounter over the follow‐up period. Our findings highlight a potential gap between clinical practice and emerging evidence that supports the use of LAI‐AP for individuals with substance use who present with psychosis for the first time. Future studies should examine clinician decision‐making and patient experiences to inform the development and implementation of strategies for recommending an LAI‐AP in diagnostically ambiguous populations.

## Funding

This work was supported by the UW Medicine Garvey Institute for Brain Health Solutions.

## Supporting information


**Table S1:** Association between diagnosis and 30‐day rehospitalisation or 30‐day ED encounter.
**Table S2:** Poisson regression model examining the association between a diagnosis of first‐episode substance‐induced psychosis and rehospitalisation during the follow‐up period (January 2021–June 2025).
**Table S3:** Sensitivity analyses including race and involuntary hospitalisation status (A) in the Cox proportional hazards models for time to rehospitalization and time to ED encounter during the follow‐up period (January 2021–June 2025), (B) in the Poisson regression models for 30‐day rehospitalization and 30‐day ED encounter.
**Table S4:** Sensitivity analyses expanding the sample to those who had a psychotic episode within the 3 years prior to their index hospitalisation (A) in the Bayesian Poisson regression model of the association between diagnosis and receiving a long‐acting injectable antipsychotic (LAI‐AP); (B) in the Cox proportional hazards models for time to rehospitalization and time to ED encounter during follow‐up; (C) in the Poisson regression models for 30‐day rehospitalization and 30‐day ED encounter.

## Data Availability

The data that support the findings of this study are available from the corresponding author upon reasonable request.
