# Supplementary material for: A Retrospective Cohort Study of Hospitalised Patients With First‐Episode Substance‐Induced Psychosis and First‐Episode Psychosis: Comparing Long‐Acting Injectable Antipsychotic Use and Post‐Discharge Outcomes
Source: Early Interv Psychiatry. 2026 Sep 21;20(9):e70250. doi: 10.1111/eip.70250 (PMC13591287; doi:10.1111/eip.70250)
Supplement: Supplementary file 1 [file EIP-20-0-s001.docx]

**Supplementary Table 1.** Association between diagnosis and 30-day rehospitalization or 30-day ED encounter.

|  | 30-day rehospitalization | | 30-day ED encounter | |
| --- | --- | --- | --- | --- |
|  | RR | 95% CI | RR | 95% CI |
| FESIP | 1.49 | 0.64-3.51 | 1.52 | 0.78-2.97 |
| LAI-AP use | 0.87 | 0.13-5.97 | 2.13 | 0.69-6.61 |
| Age | 0.97 | 0.90-1.04 | 1.00 | 0.94-1.06 |
| Male | 0.85 | 0.35-2.03 | 1.29 | 0.60-2.76 |
| Cannabis use | 1.23 | 0.44-3.39 | 1.31 | 0.58-2.94 |

Abbreviations: ED, emergency department; RR, relative risk; CI, confidence interval; FESIP, first-episode substance-induced psychosis; LAI-AP, long-acting injectable antipsychotic.

**Supplementary Table 2.** Poisson regression model examining the association between a diagnosis of first-episode substance-induced psychosis and rehospitalization during the follow-up period (January 2021-June 2025).

|  | Rehospitalization during follow-up | |
| --- | --- | --- |
|  | RR | 95% CI |
| FESIP | 1.21 | 0.70-2.08 |
| LAI-AP use | 0.85 | 0.26-2.81 |
| Age | 1.00 | 0.96-1.04 |
| Male | 1.07 | 0.61-1.86 |
| Cannabis use | 1.32 | 0.74-2.35 |

Abbreviations: RR, relative risk; CI, credible interval; FESIP, first-episode substance-induced psychosis; LAI-AP, long-acting injectable antipsychotic.

**Supplementary Tables 3.** Sensitivity analyses including race and involuntary hospitalization status **(A)** in the Cox proportional hazards models for time to rehospitalization and time to ED encounter during the follow-up period (January 2021-June 2025), **(B)** in the Poisson regression models for 30-day rehospitalization and 30-day ED encounter.

**(A)**

|  | Rehospitalization during follow-up | | ED encounter during follow-up | |
| --- | --- | --- | --- | --- |
|  | HR | 95% CI | HR | 95% CI |
| FESIP | 1.14 | 0.70-1.85 | 1.40 | 0.92-2.14 |
| LAI-AP use | 0.53 | 0.16-1.72 | 0.84 | 0.84-2.28 |
| Age | 1.01 | 0.97-1.05 | 1.02 | 0.99-1.06 |
| Male | 1.06 | 0.64-1.76 | 1.24 | 0.79-1.96 |
| Cannabis use | 1.29 | 0.77-2.15 | 1.03 | 0.33-2.15 |
| Involuntary hospitalization | 1.21 | 0.69-2.12 | 1.38 | 0.84-2.28 |
| Race (vs. Non-Hispanic White) |  |  |  |  |
| Asian | 0.70 | 0.34-1.43 | 0.61 | 0.32-1.15 |
| Black | 1.50 | 0.89-2.50 | 1.35 | 0.85-2.16 |
| Other | 0.51 | 0.20-1.33 | 0.74 | 0.37-1.51 |

**(B)**

|  | 30-day rehospitalization | | 30-day ED encounter | |
| --- | --- | --- | --- | --- |
|  | RR | 95% CI | RR | 95% CI |
| FESIP | 1.70 | 0.61-4.73 | 1.78 | 0.81-3.95 |
| LAI-AP use | 0.43 | 0.07-2.69 | 1.55 | 0.37-6.45 |
| Age | 0.99 | 0.87-1.43 | 0.97 | 0.90-1.05 |
| Male | 0.72 | 0.25-2.06 | 0.93 | 0.39-2.26 |
| Cannabis use | 0.81 | 0.25-2.68 | 0.61 | 0.23-1.58 |
| Involuntary hospitalization | 1.49 | 0.34-6.48 | 0.74 | 0.28-1.92 |
| Race (vs. Non-Hispanic White) |  |  |  |  |
| Asian | 1.74 | 0.36-8.41 | 1.62 | 0.59-4.40 |
| Black | 2.39 | 0.68-8.36 | 1.46 | 0.49-4.35 |
| Other | <0.01 | <0.01-<0.01 | <0.01 | <0.01-<0.01 |

Abbreviations: LAI-AP, long-acting injectable antipsychotic; RR, relative risk; CI, confidence interval; FESIP, first-episode substance-induced psychosis; ED, emergency department; HR, hazard ratio.

**Supplementary Tables 4.** Sensitivity analyses expanding the sample to those who had a psychotic episode within the three years prior to their index hospitalization **(A)** in the Bayesian Poisson regression model of the association between diagnosis and receiving a long-acting injectable antipsychotic (LAI-AP); **(B)** in the Cox proportional hazards models for time to rehospitalization and time to ED encounter during follow-up; **(C)** in the Poisson regression models for 30-day rehospitalization and 30-day ED encounter.

**(A)**

|  | LAI-AP use | |
| --- | --- | --- |
|  | RR | 95% CI |
| SIP3Y | 0.43 | 0.08-2.22 |
| Age | 0.98 | 0.86-1.09 |
| Male | 0.82 | 0.27-2.62 |
| Family history of psychosis | 2.40 | 0.81-6.90 |
| Cannabis use | 0.83 | 0.28-2.45 |
| Cannabis use * SIP3Y | 0.57 | 0.10-3.16 |

**(B)**

|  | Rehospitalization during follow-up | | ED encounter during follow-up | |
| --- | --- | --- | --- | --- |
|  | HR | 95% CI | HR | 95% CI |
| SIP3Y | 1.08 | 0.77-1.51 | **1.53** | **1.14-2.07** |
| LAI-AP use | 1.02 | 0.65-1.62 | 1.11 | 0.74-1.69 |
| Age | 0.99 | 0.97-1.02 | 1.00 | 0.98-1.03 |
| Male | 0.97 | 0.68-1.38 | 1.12 | 0.81-1.55 |
| Cannabis use | 0.99 | 0.71-1.40 | 0.98 | 0.72-1.32 |

**(C)**

|  | 30-day rehospitalization | | 30-day ED encounter | |
| --- | --- | --- | --- | --- |
|  | RR | 95% CI | RR | 95% CI |
| SIP3Y | 1.41 | 0.72-2.76 | 1.68 | 0.96-2.93 |
| LAI-AP use | 0.82 | 0.31-2.14 | 1.23 | 0.58-2.61 |
| Age | 0.96 | 0.90-1.03 | 0.99 | 0.94-1.04 |
| Male | 0.56 | 0.29-1.10 | 0.80 | 0. 45-1.43 |
| Cannabis use | 1.14 | 0.52-2.52 | 1.00 | 0.53-1.92 |

Abbreviations: LAI-AP, long-acting injectable antipsychotic; RR, relative risk; CI, confidence interval; SIP3Y, substance-induced psychosis diagnosed within 3 years prior to index hospitalization; ED, emergency department; HR, hazard ratio
